# The inadequacy of the ρ-T curve for phase transitions in the presence of magnetic fields

**DOI:** 10.1016/j.xinn.2025.100837

**Published:** 2025-02-10

**Authors:** Shengnan Zhang, Zhong Fang, Hongming Weng, Quansheng Wu

**Affiliations:** 1 Beijing National Laboratory for Condensed Matter Physics and Institute of Physics, Chinese Academy of Sciences, Beijing 100190, China; 2 University of Chinese Academy of Sciences, Beijing 100049, China; 3 Songshan Lake Materials Laboratory, Dongguan, Guangdong 523808, China

**Keywords:** phase transition, magnetotransport, ρ-T curve, WannierTools

## Abstract

The resistivity-temperature (*ρ(T)*) curve is traditionally employed to distinguish metallic, semiconducting, and insulating behaviors in materials, with deviations often interpreted as evidence of phase transitions. However, such interpretations are valid only under specific conditions, including the presence of a magnetic field. This study critically reexamines the *ρ(T)* curve in magnetic environments. Our findings reveal that shifts between metallic and insulating states, as well as reentrant metallic behavior, may not necessarily indicate genuine phase transitions. Instead, these phenomena can be attributed to the scaling behavior of magnetoresistance, governed by a power law dependence on both the magnetic field and temperature. Employing first-principles calculations and the Boltzmann transport method, we analyzed the magnetoresistance of SiP_2_ and NbP across varying conditions. This approach not only explains the reentrant behavior observed experimentally but also reconciles discrepancies in magnetoresistance findings reported by different research groups. These findings challenge the conventional reliance on the *ρ(T)* curve as a straightforward indicator of phase transitions in magnetic fields. We underscore the importance of accounting for standard magnetoresistance effects caused by the Lorentz force before confirming the existence of such transitions. This novel perspective advances our understanding of material properties in magnetic fields and establishes a new framework for interpreting transport phenomena in condensed matter physics.

## Public summary

• *ρ(T)* curves fail to identify phase transitions in magnetic fields.
• Scaling law and Fermi surface geometry shape *ρ(T)* curves in magnetic fields.
• Simulations match experiments for NbP and SiP_2_.
• Magnetotransport reveals intrinsic electronic structures.
• WannierTools calculates magnetotransport of real materials beyond the dirty limit.

## Graphical abstract

**Figure:**
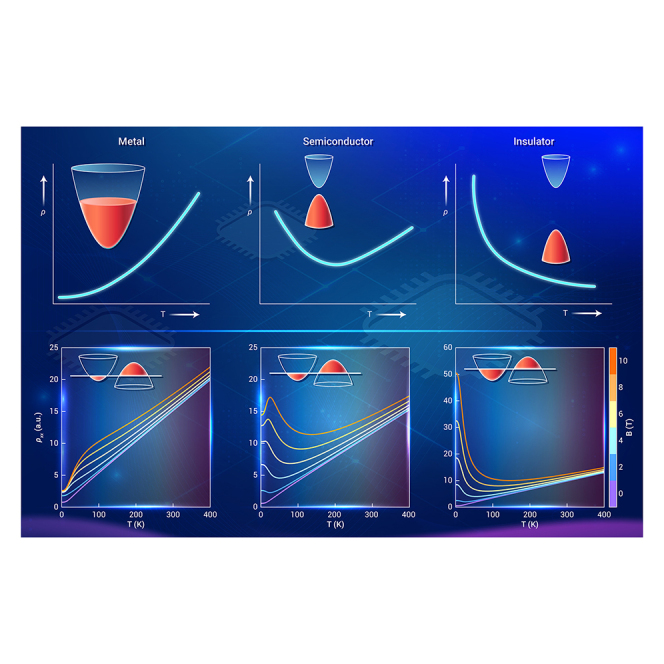

## Introduction

The resistivity-temperature curve, ρ(T), has traditionally served as an empirical criterion for determining the phase of a material in solid-state physics.^1^^,^^2^ Metals typically exhibit an increase in resistivity with rising temperature, while insulators show a decrease, and semiconductors demonstrate a decrease at low temperatures followed by an increase at higher temperatures. At the turn of the century, theoretical and experimental investigations of graphite and bismuth spurred detailed studies of their temperature-dependent magnetotransport properties.^3^^,^^4^^,^^5^^,^^6^^,^^7^^,^^8^^,^^9^^,^^10^^,^^11^^,^^12^^,^^13^ Notably, Kopelevich et al. observed reentrant metallic behavior in graphite, which they suggested could be associated with the quantum Hall effect and superconducting correlations.^6^ In 2005, Du et al. demonstrated that the magnetic field-induced metal-insulator transition in graphite and bismuth was driven by the unique alignment and spacing of three characteristic energy scales.^9^

The discovery of extreme magnetoresistance (XMR) in ${\text{WTe}}_{2}$ in 2014 reignited interest in magnetoresistance (MR) and magnetic field-induced metal-insulator transitions.^14^ A 2015 study revisiting Kohler’s rule provided insights into why resistivity, under the influence of a magnetic field, mimics characteristics of metal-insulator transitions as temperature changes. This phenomenon, referred to as the “upturn phenomenon,” has since been observed in numerous materials exhibiting XMR.^15^^,^^16^^,^^17^^,^^18^^,^^19^^,^^20^^,^^21^^,^^22^^,^^23^^,^^24^^,^^25^^,^^26^^,^^27^^,^^28^^,^^29^^,^^30^^,^^31^^,^^32^ In 2017, reentrant metallic behavior was reported in the Weyl semimetal NbP,^17^ where semiclassical theory successfully explained these anomalies without invoking exotic mechanisms. The dependence of MR on magnetic field and temperature is highly complex and currently lacks a comprehensive theoretical framework to identify dominant mechanisms and correlate them with experimental observations. In this study, we systematically investigated the scaling behavior of resistivity under varying magnetic fields and temperatures. Utilizing the two-band model, we analyzed MR behaviors in representative materials such as NbP and ${\text{SiP}}_{2}$, elucidating the phenomena of “reentrant metallic” and “metal insulator-like” transitions.

## Materials and methods

To calculate the magnetotransport properties of real materials, we employed a combination of first-principles calculations and semiclassical Boltzmann transport theory. Tight-binding models were constructed using first-principles software such as Vienna Ab initio Simulation Package (VASP)^33^^,^^34^ and Wannier function techniques implemented via Wannier90.^35^ Detailed computational methods have been described previously^36^^,^^37^ and were implemented with the WannierTools software package.^38^

## Results and discussion

MR is directly related to the distance carriers travel before scattering, quantified by the number of orbits completed on the Fermi surface.^1^^,^^39^ This relationship is expressed by the parameter $\omega \tau =\frac{eB\tau }{m}$, where m is the cyclotron mass. This principle is supported by the Chambers equation,^39^ which expresses MR as a function of the product of the magnetic field B and relaxation time τ (see supplemental information):

(Equation 1) $$\text{MR}=\frac{\rho \left(B\right)-\rho_{0}}{\rho_{0}}\propto {\left(B\tau \right)}^{\gamma }={\left(\frac{B}{\alpha \rho_{0}}\right)}^{\gamma }$$

where τ is approximated as $\tau =1/\alpha \rho_{0}$, with $\rho_{0}$ representing the temperature-dependent resistivity at B=0, and $\alpha =ne^{2}/m$, where n and m denote the carrier density and effective mass, respectively. The longitudinal resistivity ρ(B,T) can then be expressed as follows:

(Equation 2) $$\rho \left(B,T\right)=\rho_{0}\left(T\right)+\zeta \frac{B^{\gamma }}{{\left(\alpha \rho_{0}\right)}^{\gamma -1}}$$

where ζ is a material-dependent constant. The exponent γ in the power law dependence of MR on the magnetic field is particularly critical, as it determines the potential competition between the first and second terms on the right side of Equation 2. This interplay leads to a reversal of the temperature dependence of resistivity beyond a certain magnetic field strength. To extract the underlying physics, we adopted Kohler’s rule, but our conclusions remained robust, as explained in the article.

**Figure 1:**
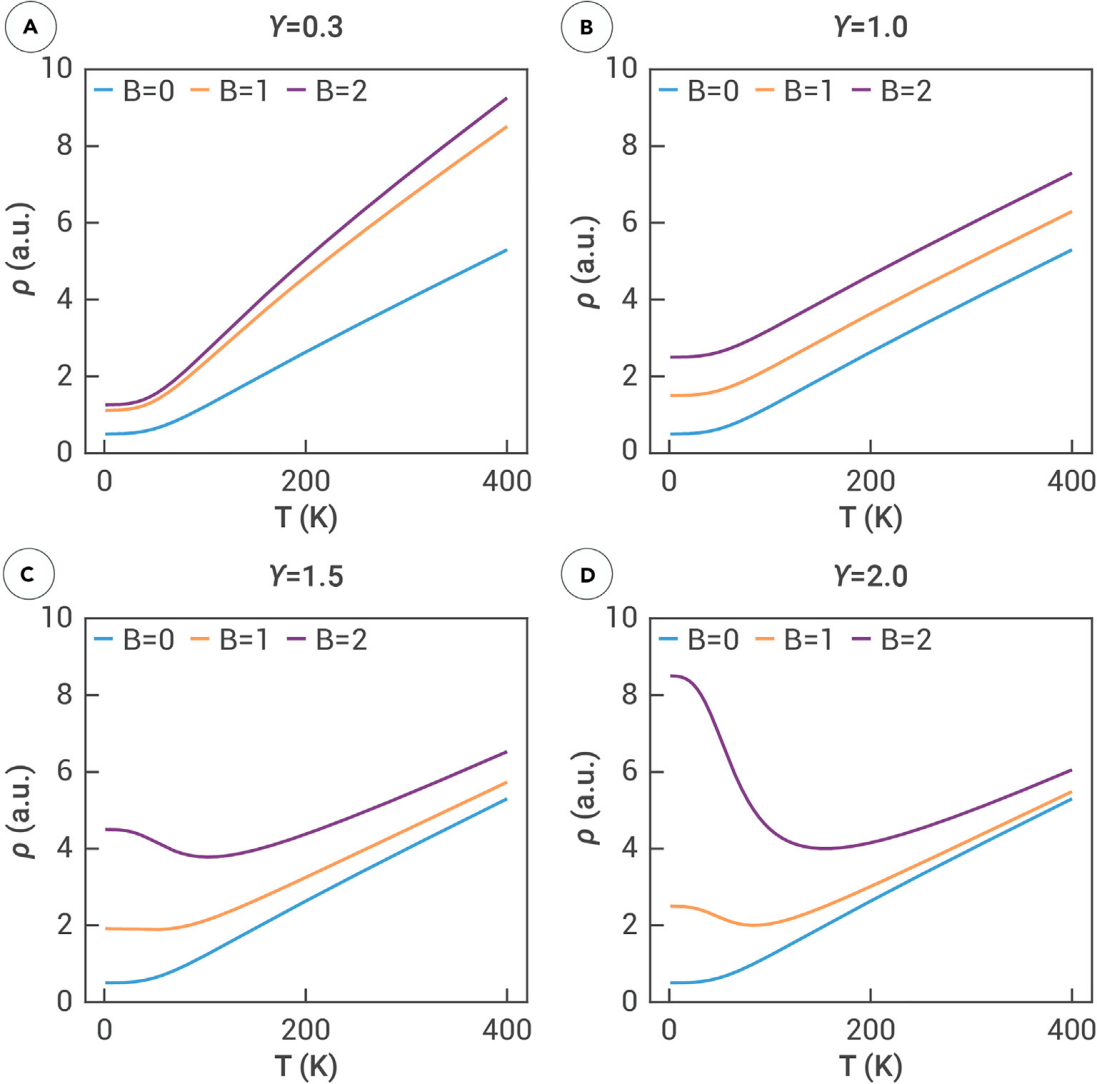
Temperature dependent resistivity at different magnetic fields for different exponents γ (A) *γ* = 0.3, (B) *γ* = 1.0, (C) *γ* = 1.5, (D) *γ* = 2.0.

To begin, we consider the simplest case, γ=1, where the second term on the right side of Equation 2 stabilizes to a constant. Under these conditions, the expression for ρ(B,T) can be represented as $\rho =\rho_{0}\left(T\right)+\text{const}$. Consequently, ρ precisely inherits the temperature dependence of $\rho_{0}$, adjusted by a constant shift. This behavior can be visualized as a series of ρ(T) curves evenly spaced across different temperatures and magnetic fields, as illustrated in Figure 1B. Note that the zero-field resistivity $\rho_{0}$ was calculated using the Bloch-Grüneisen model throughout this study.^2^^,^^40^^,^^41^^,^^42^

For the case where γ<1, both terms on the right side of Equation 2 remain proportional to $\rho_{0}$. Therefore, the longitudinal resistivity ρ(T) continues to exhibit a consistent increase with temperature, mirroring $\rho_{0}\left(T\right)$. However, the rate of increase in the second term differs from that of the first, resulting in a series of curves that are unevenly spaced, as depicted in Figure 1A for γ=0.3. This behavior contrasts with the evenly spaced curves observed in the γ=1 case.

When the exponent γ>1, the situation becomes more complex. The opposing temperature dependencies of the two terms in Equation 2 result in a competition that introduces a turning point in the ρ(T) curves. For example, the ρ(T) curves for B=1 and B=0, as shown in Figure 1D (γ=2), exhibit distinctly different behaviors at low temperatures. For B=0, the resistivity ρ consistently rises with temperature. However, at B=1, the resistivity initially decreases in the low-temperature range before increasing, with this behavior becoming more pronounced at B=2. This phenomenon is the underlying origin of the metal-insulator-like behavior discussed in Wang et al.^43^

The reason for plotting both γ=1.5 and γ=2 is to observe the increasing prominence of the resistivity reversal trend with temperature as γ increases. This observation confirms that metal-insulator-like characteristics in the ρ(T) curves are more readily detectable experimentally when charge carriers are close to perfect compensation. Notably, the curves for γ<1 and γ>1 deviate from the equidistant spacing observed in the γ=1 scenario due to the influence of the second term $\frac{B^{\gamma }}{{\left(\alpha \rho_{0}\right)}^{\gamma -1}}$ in Equation 2. For γ<1, the spacing between the curves diminishes as the magnetic field increases (due to the diminishing rate of $B^{\gamma }$) but expands as the temperature increases (since the rate of $\rho_{0}^{1-\gamma }$ accelerates). Conversely, for γ>1, the spacing widens with an increasing magnetic field (due to the rapid growth of $B^{\gamma }$) but narrows as the temperature rises (due to the decreasing rate of $\rho_{0}^{1-\gamma }$).

The three scenarios discussed above, γ<1, γ=1, and γ>1, represent the most fundamental dependencies of ρ(B). However, in real materials, the power law exponent γ is not fixed; it varies with temperature, orientation, and magnetic field strength. As a result, the behavior of the ρ(T) curve exhibits more complex characteristics.

To further analyze these variations, we employed a two-band model to study the temperature-dependent resistivity of charge carriers under different degrees of compensation: (A) $n_{e}=0.3$ and $n_{h}=1$, (B) $n_{e}=0.7$ and $n_{h}=1$, C $n_{e}=1$ and $n_{h}=1$. These cases produce three distinct field-dependent ρ(T) curves, resistivity kink behavior, reentrant metallic behavior, and metal-insulator-like transition behavior.

### Resistivity kink behavior

**Figure 2:**
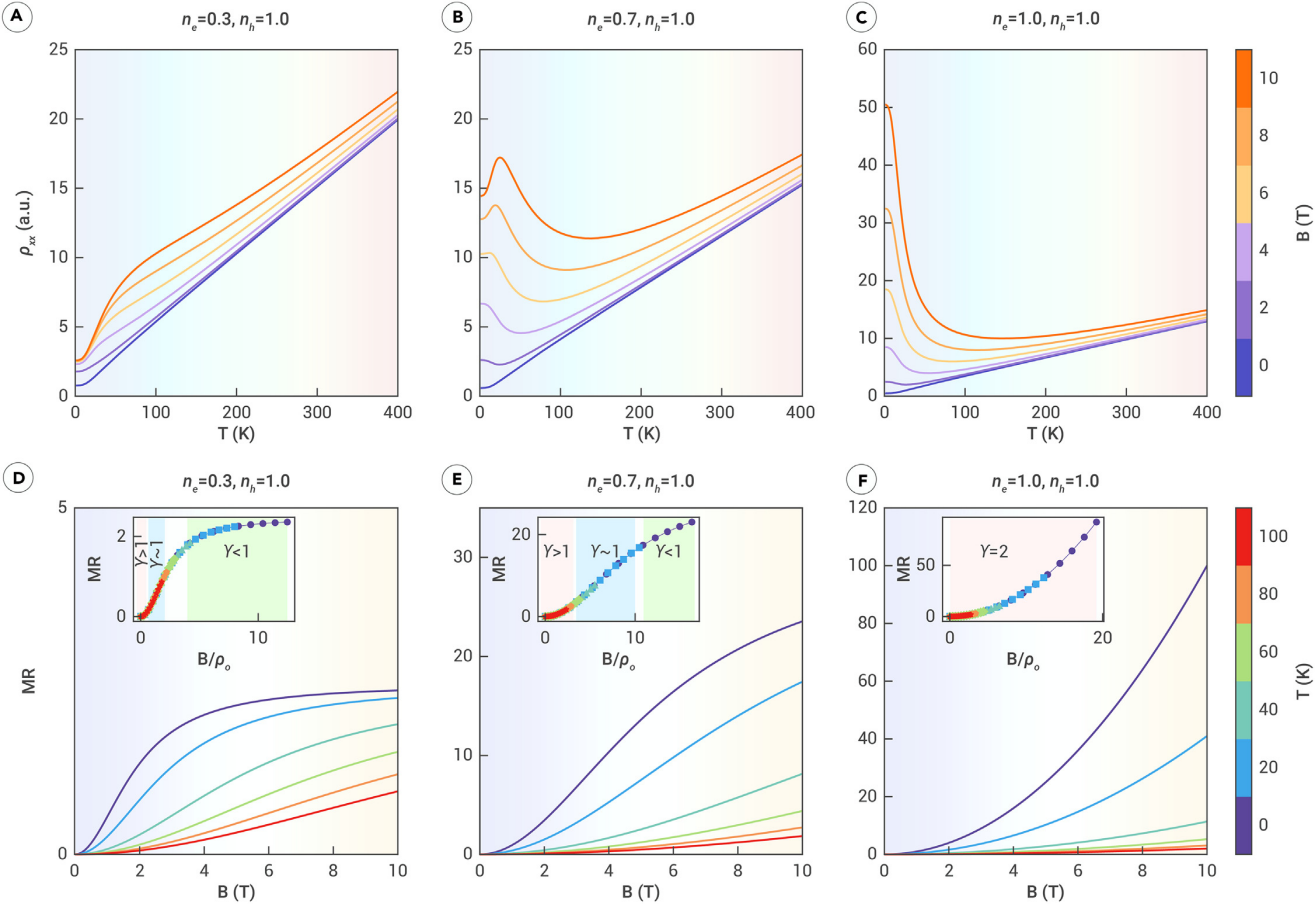
Temperature- and field-dependent resistivity at different exponents γ Shown are (A–C) the temperature-dependent ρ(T) and (D–F) The field-dependent MR(B). The color of the ρ(MR) curve matches the color bar corresponding to the magnetic field B (temperature T). The background color of the ρ(MR) graphic indicates the numerical values of temperature T (field strength B), representing areas of low temperature (low field), intermediate temperature (intermediate field), and high temperature (high field). Insets in (D)–(F) show the scaled $\text{MR}\left(B/\rho_{0}\right)$ curves at different temperatures.

We first discuss the weak compensation case, where $n_{e}=0.3$ and $n_{h}=1$ represent the electron and hole concentrations, respectively. In Figure 2A, the ρ(T) curves can be categorized into three temperature zones: low temperature (0–60 K), intermediate temperature (80–200 K), and high temperature (200 K and above). The inset in Figure 2D confirms that the region where γ≥1 is quite limited, as most curves concentrated in the MR saturation phase (γ<1). These curves are further segmented based on temperature and magnetic field. The low-temperature, high-field area corresponds to γ<1, as observed in the low-temperature region of Figure 2A, where ρ(T) rises monotonically with temperature. This is indicated by the color gradient transitioning from pink to red. The progression of these curves resembles the behavior depicted in Figure 1A.

The inset in Figure 2D shows that, as the temperature reaches the intermediate range (80–200 K), MR(B) approximates a linear relationship, as indicated by the light-blue-shaded region. This corresponds to the γ=1 case depicted in Figure 1A. In this range, the curves in Figure 2A transition from light purple to orange, maintaining an approximately equal spacing similar to the characteristics in Figure 1B. When the temperature enters the high-temperature zone (200 K and above), the MR dependence on the magnetic field exceeds the previous linear trend, as depicted by the light pink-shaded region in the inset in Figure 2D. In this high-temperature range, the color transition from dark purple to light purple to orange in Figure 2A illustrates the increasing spacing between the curves, resembling the high-temperature behavior shown in Figure 1C. Thus, in the case of weak compensation, the behavior of ρ(T) can be comprehensively understood through the patterns and transitions observed in Figure 1.

### Reentrant behavior

We now consider the close-to-compensation case, exemplified by $n_{e}=0.7$ and $n_{h}=1$. Compared to Figure 2D, the area where γ≥1 in the inset of Figure 2E is significantly larger, indicating notable changes in the intermediate temperature and magnetic field regions. The resistivity behaves similarly in Figure 2A in the low-temperature and high-field region (i.e., γ<1): ρ(T) rises with increasing temperature. This is depicted by the yellow to orange curves in Figure 2B.

However, as the magnetic field increases, the exponent of MR(B) in Figure 2E transitions to γ>1, introducing a new feature in ρ(T): a decrease in resistivity with increasing temperature. This behavior is illustrated by the light dark purple to light purple curves in the low-temperature region in Figure 2B. As the magnetic field increases further, this feature becomes more pronounced because the exponent approaches γ=2 at slightly higher temperatures, as shown by the yellow to orange curves in Figure 2B. In the high-temperature region, where γ>1 persists, the ρ(T) curves replicate the behavior observed in Figure 1D. A similar ρ(T) curve at B=10 has been reported in both graphite^6^^,^^10^^,^^11^^,^^12^ and NbP,^17^ where it is referred to as “reentrant metallic behavior” by the respective authors. This reentrant metallic behavior is one of the most important conclusions of our study. Unlike prior work relying on simplified models, we systematically analyzed this phenomenon using the most fundamental Chambers equation,^39^ supported by rigorous calculations.

### Metal-insulator-like transition

Finally, we consider the ideal case where electron-hole carriers are perfectly compensated. In this scenario, the MR(B) consistently follows a quadratic scaling with γ=2 regardless of changes in magnetic field and temperature (see inset in Figure 2F). Consequently, ρ(T) in Figure 2C precisely replicates the characteristics shown in Figure 1D. While perfect compensation is rare in real materials, most materials exhibiting large MR are nearly perfectly compensated, as shown in Figure 1C. This ensures that experimentally observed ρ(T) curves often resemble those in Figure 2C.

**Figure 3:**
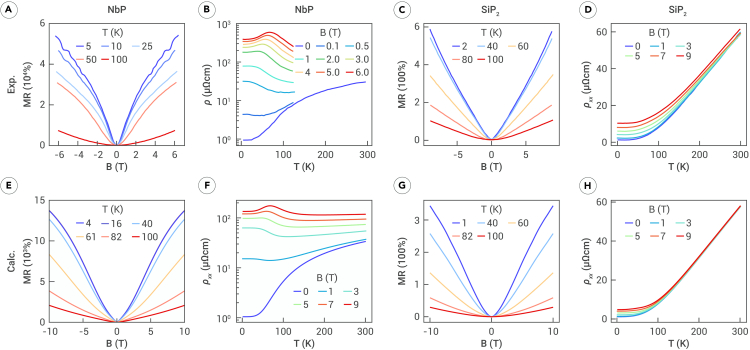
Experimentally observed and theoretically calculated temperature-dependent MR and resistivity of NbP and ${\text{SiP}}_{2}$ (A and B) Reprinted from Sudesh Kumar et al.^44^ under the Creative Commons Attribution 4.0 International License. (C and D) Reprinted from Zhou et al.^23^ (E and F) The calculated MR and resistivity of NbP following electron doping (shifting the Fermi level by 7 meV). (G and H) The calculated MR and resistivity of ${\text{SiP}}_{2}$ when the magnetic field is applied along the z direction.

From this analysis, it is clear that the shape of the MR(B) curve plays a critical role in determining the ρ(T) curve. However, the reentrant metallic behavior depicted in Figure 2B is rarely observed in experiments measuring temperature-dependent resistivity. This is because the conditions for observing a peak in the ρ(T) curve are stringent. Specifically, the MR(B) curve must exhibit a γ<1 phase to ensure an increase in resistivity at low temperatures and a γ>1 phase to facilitate a decrease in resistivity with increasing temperature. Experimental data satisfying these criteria are limited. To validate our findings, we computed and analyzed the MR and resistivity of two real materials: NbP and ${\text{SiP}}_{2}$. Figure 3, top, displays experimental measurements for NbP and ${\text{SiP}}_{2}$ from previous studies,^23^^,^^44^ respectively, while the bottom presents numerical simulation results. Both materials showed good agreement between experimental data and simulations. Details of the calculations are provided in the supplemental information.

### Real material NbP

NbP, a typical Weyl semimetal,^45^^,^^46^ has been observed by several experimental groups to exhibit a peak in the low-temperature ρ(T) curves,^17^^,^^44^^,^^47^ resembling the peak shown in Figure 2B. This phenomenon, referred to as reentrant metallic behavior,^6^^,^^17^ is depicted in Figures 3A and 3B, which show the MR(B) and ρ(T) curves derived from Sudesh Kumar et al.^44^ At low temperatures, the field dependent MR of NbP transitions from parabolic to linear. Above 100 K, the MR dependence reverts to a parabolic form. This behavior aligns precisely with the conditions for reentrant metallic behavior discussed in relation to Figure 2B. As shown in Figure 3B, the ρ(T) curve exhibits the expected peak. The theoretical calculations in Figures 3E and 3F successfully reproduce these experimental observations, validating our theoretical framework. Similar phenomena have been observed in TaP, consistent with these findings, though they are not elaborated upon here. It is worth noting that the chemical potential of specific NbP samples is critical for observing reentrant behavior. Our theoretical calculations indicate that clean samples lead to metal-insulator-like behavior, as shown in Figure S3, while doped samples exhibit reentrant behavior.

### Real material $\text{Si}P_{2}$

${\text{SiP}}_{2}$, a topologically trivial semimetal with highly anisotropic Fermi surfaces, as shown in Figure S2, exhibits MR(B) scaling behavior that depends on the orientation of the magnetic field. For brevity, we focus on the case of B‖z axis (θ=0). In this configuration, ρ(B) is approximately linear (γ approximately 1.2) at low temperatures and quadratically dependent on B at higher temperatures, as shown in Figure 3C and 3G. The ρ(T) curves for different magnetic fields, illustrated in Figure 3D and 3H, exhibit monotonically increasing behavior with temperature. These curves do not precisely replicate Figure 1B due to γ≠1 for ${\text{SiP}}_{2}$, resulting in unevenly spaced curves. The spacing increases with the magnetic field, consistent with the curve spacing analysis in Figure 1D. Furthermore, because MR(B) deviates only slightly from linearity, there is no distinct decline followed by an increase, as observed in Figures 1C and 1D. As anticipated, the calculated results for ${\text{SiP}}_{2}$ closely match the experimental data, further validating the theoretical approach. In the supplemental information, we further explore the scenario in which the magnetic field is oriented along the *yz* direction (*θ* = 45°). Under these conditions, the MR exhibits nearly quadratic, unsaturated behavior, and theoretical and experimental results—which show excellent agreement—indicate a metal-insulator-like transition, as shown in Figure S2, consistent with our earlier model analysis.

Finally, it is important to emphasize that, while our analysis is based on the assumption of Kohler’s rule, the conclusions are universally valid and can be extended to systems that deviate from Kohler’s rule. Kohler’s rule may fail when the relaxation times of multiple carriers differ significantly and lack common factors. We postulate that the electron and hole carriers vary with temperature, as shown in Figure S1. Using this assumption, we plotted the MR(B) and ρ(T) curves for the system in Figure S1. Despite minor discrepancies, the overall behavior and trends of MR(B) and ρ(T) remain consistent with scenarios where Kohler’s rule holds.

## Conclusion

In this work, we comprehensively calculated and explained the resistivity kink, reentrant metallic, and metal-insulator-like transition behaviors within a unified framework. We show that these complex ρ(T) behaviors under a magnetic field are primarily governed by the material’s intrinsic electronic structure and doping concentration, with the geometry of Fermi surfaces playing a pivotal role in the diffusive transport regimen. The ρ(T) curves for different materials can be derived from three fundamental scaling behaviors of MR(B) as γ<,=,>1. We then applied our theory to real materials, such as NbP and ${\text{SiP}}_{2}$, analyzing their resistivity under varying magnetic fields and temperatures. Our calculations showed excellent agreement with experimental measurements. These findings suggest that magnetic field effects can be scaled similarly as temperature effects when studying resistivity. Therefore, the ρ(T) curve cannot be reliably used as standalone evidence of a phase transition in the presence of a magnetic field.

## Acknowledgments

This work was supported by the 10.13039/501100012166National Key R&D Program of China (2023YFA1607400 and 2022YFA1403800), the 10.13039/501100001809National Natural Science Foundation of China (12274436, 11925408, and 11921004), and the Science Center of the National Natural Science Foundation of China (12188101), and H.W. acknowledges support from the New Cornerstone Science Foundation through the XPLORER PRIZE.

## Author contributions

Q.W. designed and conceived the overall project. S.Z. performed the calculations. S.Z. and Q.W. drafted the manuscript. All authors contributed to data analysis and manuscript refinement.

## Declaration of interests

The authors declare no competing interests.

## Supplemental information

Document S1. Figures S1–S3

Document S2. Article plus supplemental information

## Footnotes

It can be found online at https://doi.org/10.1016/j.xinn.2025.100837.
