# Supplementary material for: The inadequacy of the ρ-T curve for phase transitions in the presence of magnetic fields
Source: Innovation (Camb). 2025 Feb 10;6(5):100837. doi: 10.1016/j.xinn.2025.100837 (PMC12105513; doi:10.1016/j.xinn.2025.100837)
Supplement: Document S1. Figures S1–S3 [file mmc1.pdf]

The Innovation, Volume *6*

Supplemental Information

The inadequacy of the ρ-T curve for phase transitions in the presence of magnetic fields

Shengnan Zhang, Zhong Fang, Hongming Weng, and Quansheng Wu

Abstract

This supplementary material provides a detailed discussion on the conditions for the validity of Kohler's rule. It also demonstrates that even if Kohler's rule does not hold, our conclusions regarding the magnetic field dependence of the ρ-T curve remain valid. Additionally, it presents methods for calculating magnetoresistance using first-principles and Boltzmann theory, discusses how to combine theoretical and experimental data to fit the temperature dependence of the relaxation time, and includes more detailed calculations for the SiP2 and NbP materials.

The derivation of Kohler's rule

Given there are more than one type of charge carrier, the relaxation time of which are distinct with each other, thus the Kohler's rule could be invalid. However, there are exceptions, such as different charge carriers with totally the same relaxation time or common factor, i.e., $\tau_h = \lambda\tau_e = \lambda\tau$, where λ is a constant and independent of temperature, then one could write the total longitudinal resistivity as following,

$$\rho_{xx} = \frac{(\frac{n_e\tau_e}{m_e} + \frac{\lambda n_h\tau_e}{m_h}) + (\frac{\lambda n_e\tau_e}{m_h} + \frac{n_h\tau_e}{m_e})\frac{\lambda e^2\tau_e\tau_e}{m_e m_h}B^2}{(\frac{en_e\tau_e}{m_e} + \frac{\lambda en_h\tau_e}{m_h})^2 + (n_h - n_e)^2\frac{\lambda^2 e^4\tau_e^2\tau_e^2}{m_e^2 m_h^2}B^2}$$
$$= \frac{(\frac{n_e}{m_e} + \frac{\lambda n_h}{m_h})\tau_e + (\frac{\lambda n_e}{m_h} + \frac{n_h}{m_e})\frac{\lambda e^2\tau_e^3}{m_e m_h}B^2}{(\frac{en_e}{m_e} + \frac{\lambda en_h}{m_h})^2\tau_e^2 + (n_h - n_e)^2\frac{\lambda^2 e^4\tau_e^4}{m_e^2 m_h^2}B^2}$$

we multiply a relaxation time on both side and get,

$$\rho_{xx}\tau = \frac{(\frac{n_e}{m_e} + \frac{\lambda n_h}{m_h}) + (\frac{\lambda n_e}{m_h} + \frac{n_h}{m_e})\frac{\lambda e^2}{m_e m_h}(B\tau)^2}{(\frac{en_e}{m_e} + \frac{\lambda en_h}{m_h})^2 + (n_h - n_e)^2\frac{\lambda^2 e^4}{m_e^2 m_h^2}(B\tau)^2} \tag{S1}$$

From the above equation, it's easy to tell that $\rho_{xx}\tau$ is a function of $B\tau$ together instead of B and τ independently.

By defining ρ_0 the resistivity at $B = 0$:

$$\rho_0\tau = \frac{m_e m_h}{e^2(n_e m_h + \lambda n_h m_e)} = \frac{1}{\alpha} \tag{S2}$$

where $\alpha = \frac{e^2(n_e m_h + \lambda n_h m_e)}{m_e m_h}$.

Eq.(S1) can be rewritten as

$$\rho_{xx}\tau = \rho_0\tau + f(B\tau) \tag{S3}$$

where $f(B\tau)$ is function of $B\tau$. By replacing τ with $\frac{1}{\alpha\rho_0}$, Eq.(S3) becomes

$$\frac{\Delta\rho_{xx}}{\rho_0} = \frac{\rho_{xx} - \rho_0}{\rho_0} = f(B/\rho_0) \tag{S4}$$

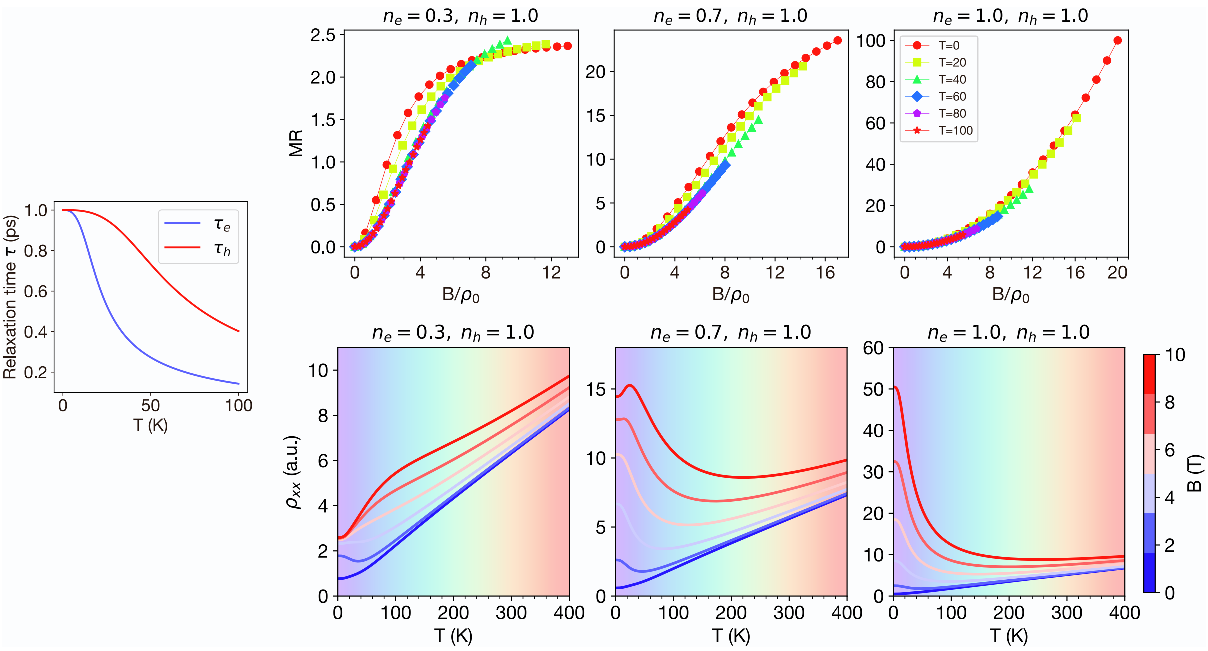

Figure S1: Violation of Kohler's rule occurs when the temperature dependence of the relaxation times for two carriers differs, such that $\tau_e/\tau_h \neq Const$. TThe upper row displays the scaled MR curves at various temperatures, while the bottom row shows $\rho(T)$ at different magnetic fields. Although Kohler's rule is violated, the complex resistivity patterns are still observable.

which is the Kohler's rule that we are familiar.

In the main text, we use only one relaxation time, where Kohler's rule is satisfied, to discuss the scaling rule; this simplifies the explanation of complex field- and temperature-dependent resistivities. However, In Figure S1, we demonstrate that even though Kohler's rule is violated, the scaling behavior can still be used to explain the complex resistivity behaviors.

Kohler's rule and Chambers equation

Kohler's rule[1] states that the MR can be described as a function of the ratio between magnetic field B and zero-field resistivity ρ_0, i.e., B/ρ_0. This rule can be proved using the Chambers equation[2,3] when all carriers have the same temperature dependent relaxation time. Chamber equation shows that the resistivity times relaxation time $\rho\tau$ is a function of the combination of magnetic field strength and the relaxation time $B\tau$, i.e. $\rho\tau = f(B\tau)$.

$$\mathrm{MR} = \frac{\rho(B) - \rho_0}{\rho_0} \propto (B\tau)^\gamma = (\frac{B}{\alpha\rho_0})^\gamma \tag{S5}$$

where the relaxation time τ is approximated as $\tau = 1/\alpha\rho_0$ with ρ_0 is the temperature dependent resistivity at $B = 0$.

Magnetoconductivity calculations

The band resolved conductivity tensor in presence of the magnetic field can be obtained by solving the Boltzmann transport equation within the relaxation time approximation as[3],

$$\frac{\boldsymbol{\sigma}^{(n)}(\boldsymbol{B})}{\tau_n} = \frac{e^2}{\alpha\pi^3}\int d\boldsymbol{k}\boldsymbol{v}_n(\boldsymbol{k})\overline{\boldsymbol{v}}_n(\boldsymbol{k})\left[-\frac{\partial f}{\partial\varepsilon_n(\boldsymbol{k})}\right], \tag{S6}$$

where α is a spin degeneracy related number, $\alpha = 4(8)$ if spin-orbit coupling is excluded (included) in the Hamiltonian, n is the band index, f is the Fermi-Dirac distribution. $\varepsilon_n(\boldsymbol{k})$, τ_n and $v_n(\boldsymbol{k})$ are the eigenvalue, relaxation time and group velocity of the n-th band, respectively. This equation is also known as the Chamber's formula[2]. The $\overline{\boldsymbol{v}}_n(\boldsymbol{k})$ describes the weighted average velocity during the past trajectory of the charge carriers ,

$$\overline{\boldsymbol{v}}_n(\boldsymbol{k}) = \int_{-\infty}^{0}\frac{dt}{\tau_n}e^{\frac{t}{\tau_n}}\boldsymbol{v}_n\left[\mathbf{k}(t)\right]. \tag{S7}$$

The orbital motion of charge carriers in the reciprocal space follows the semiclassical equation of motion,

$$\hbar\dot{\boldsymbol{k}} = -e\boldsymbol{v}_n(\boldsymbol{k})\times\boldsymbol{B}, \tag{S8}$$

where the driven force of electric field was dropped off since we are use the linear response theory on electric field[3]. The resistivity tensor is obtained by directly inverse of the conductivity tensor

$$\boldsymbol{\rho} = \boldsymbol{\sigma}^{-1} \tag{S9}$$

There are two ways in which temperature can affect the resistivity tensor in this method. One is through Fermi's distribution, $f(\varepsilon_n(\mathbf{k}))$, and the other is through the temperature-dependent relaxation time, $\tau(T)$. In metals and many semimetals with very large Fermi surfaces, the high density of charge carriers means that temperature seldom affects this density. In such cases, $\tau(T)$ primarily influences $\rho(T)$, highlighting its role in the temperature-related effects on resistivity. So next we are going to discuss how we roughly fit the relaxation time to the experiments.

Fitting $\tau(T)$ with experiments: Determining the relaxation time is a challenging task due to many unknown factors such as the cleanliness of the sample, the scattering mechanisms involved, and so on. It is well-known that the relaxation time is a reasonable approximation for most materials if the system is away from the phase transition point, away from the quantum scattering region, and not subject to large fluctuations[3]. In the Drude model, the zero-field resistivity $\rho_0 = 1/ne\mu = m^*/ne^2\tau$, where $m*$ is a effective mass, leads to $\tau = m^*/ne^2\rho_0$. As mentioned previously, in metals and some semimetals, the large Fermi surfaces ensure that temperature seldom affects the density,allowing us to assume that $\tau(T) \propto 1/\rho_0(T)$, where $\rho_0(T)$ is the measured resistivity at zero field. Usually, the $\rho_0(T)$ can be by the Bloch-Grüneisen model[4] as,

$$\rho_{sc}(T) = \rho_0 + \alpha\left(\frac{T}{\Theta_R}\right)^n\int_0^{\frac{\Theta_R}{T}}\frac{x^n}{(e^x-1)(1-e^{-x})}dx, \tag{S10}$$

with four parameters ρ_0, α, n, and Θ_R can be obtained by fitting the experiment data.

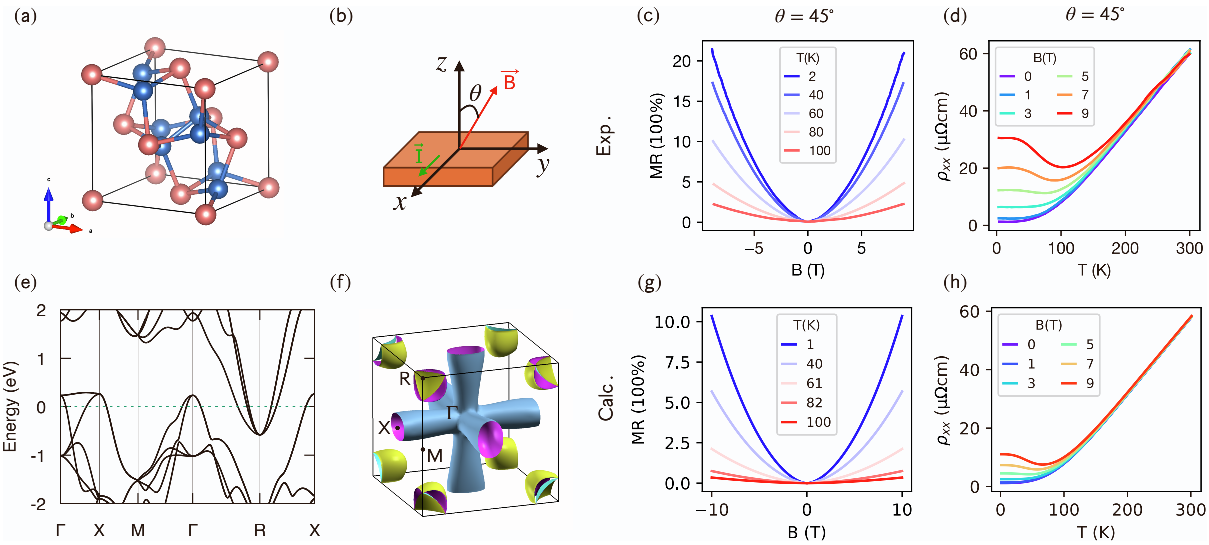

Figure S2: (a) Crystal structure with lattice constants a=b=c=5.7045 Å; (e) band structure and (f) Fermi surface of SiP_2; (b) sketch of the experimental setup. (c) and (d) show the experimentally measured field-dependent magnetoresistance MR(B) and temperature-dependent longitudinal resistivity $\rho(T)$, respectively, when the magnetic field is oriented 45° away from the z-axis in the yz-plane. (g) and (h) provide the numerically simulated MR(B) and $\rho(T)$ corresponding to (c) and (d), respectively.

By using the Chamber's formula, we can calculate the quantity $\rho\tau$ as a function of $B\tau$. To determine the relaxation time τ_0 at the Zero magnetic field, we can use the calculated value of $\rho\tau$ at $B\tau = 0$ and divide it by the experimentally measured ρ_0:

$$\tau_0 = \frac{\rho\tau(B\tau = 0)|_{calc.}}{\rho_0|_{exp.}} \tag{S11}$$

More calculations on SiP_2 and NbP

SiP_2: The calculated band structure and the Fermi surfaces are shown in Figure S2. In the main text, we discuss only the magnetoresistance when the magnetic field is aligned along the z direction $\theta = 0$, where the MR is almost linearly dependent on B. This leads to the equally spaced $\rho(T)$ values as the magnetic field changes. In Ref.[5], the experimental group also measured the magnetoresistance when the field is oriented 45° away from the z-axis in the yz-plane. As shown in Figure S2 (c), the MR(B) scales almost quadratically with B, which leads to the "metal-insulator-like" behavior, as shown in Figure S2 (d). Our numerical simulations, as presented in Figure S2(g) and (h), match the experimental observations quite well.

NbP: There are two kinds of experimental results on the resistivity behavior in NbP: one is the "reentrant-metallic" behavior discussed in the main text, and the other is the "metal-insulator-like" behavior. The latter usually shows larger MR effects at the same temperature and magnetic field strength. Our numerical simulations show that a clean sample brings the chemical potential close to the charge neutral point, allowing for compensation between electrons and holes. In this case, the MR scales quadratically with

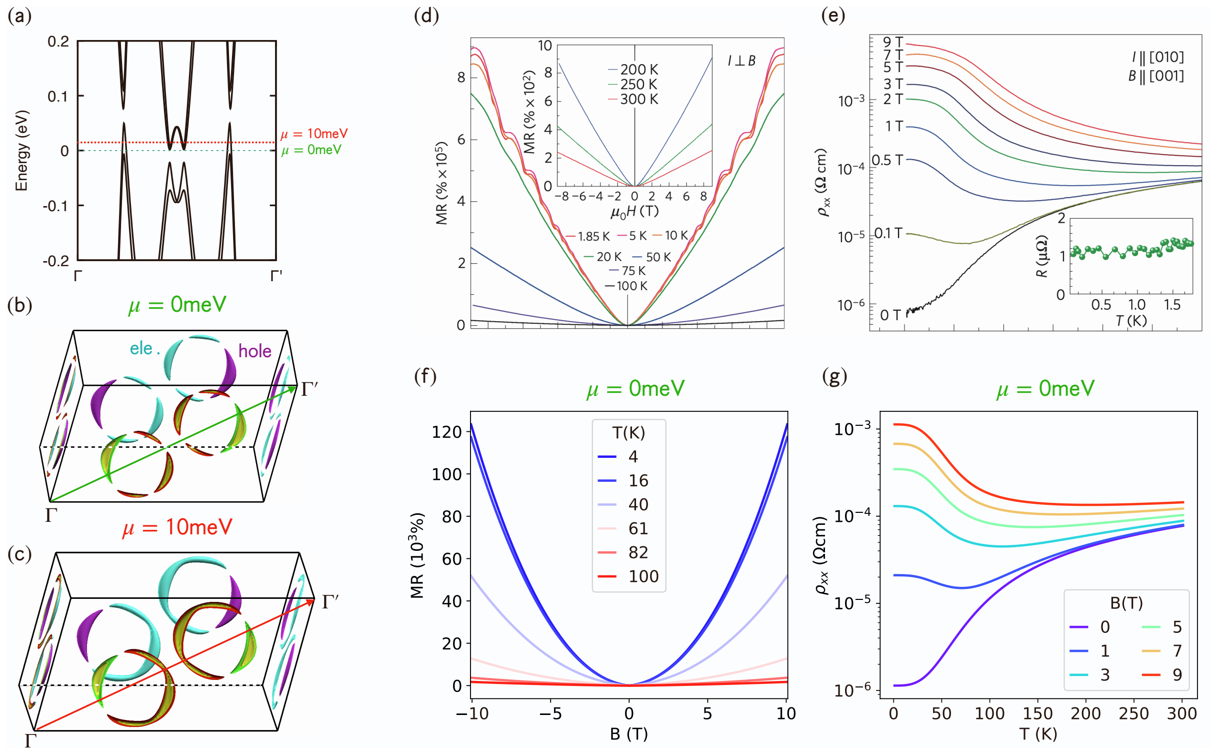

Figure S3: (a) Band structure of Weyl semimetal NbP, (b) Fermi surface of NbP at the charge neutral point, (c) Iso-energy surface at chemical potential $\mu = 10meV$, (d) and (e) are the experimental measured MR(B) and $\rho(T)$ from Ref. , (f) and (g) are the numerical calculated MR(B) and $\rho(T)$ of NbP.

B, eventually leading to the "metal-insulator-like" behavior. The comparison between the experiments (panels (d) and (e)) and our numerical simulations (panels (f) and (g)) is shown in Figure S3. The match is quite good. In the main text, we demonstrate that doping (for example,$\mu = 10meV$) causes the system to deviate from this compensation, eventually leading to the "reentrant-metallic" behavior.

References

1. Kohler, M. (1938). Zur magnetischen Widerstandsänderung reiner Metalle. *Annalen der Physik* **424**:211. DOI: 10.1002/andp.19384240124.
2. Chambers, R. G. (1952). The Kinetic Formulation of Conduction Problems. *Proc. Phys. Soc. A* **65**:458. DOI: 10.1088/0370-1298/65/6/114.
3. Ashcroft, N. W. and Mermin, N. D. (1976). *Solid State Physics*. Thomson Learning.
4. Ziman, M. (1962). *Electrons and Phonons*. Clarendon Press.
5. Zhou, Y., Lou, Z., Zhang, S., et al. (2020). Linear and quadratic magnetoresistance in the semimetal SiP_2. *Phys. Rev. B* **102**:115145. DOI: 10.1103/PhysRevB.102.115145.
